# Identification of A Putative T6SS Immunity Islet in Salmonella Typhi

**DOI:** 10.3390/pathogens9070559

**Published:** 2020-07-11

**Authors:** Luke A. F. Barretto, Casey C. Fowler

**Affiliations:** Department of Biological Sciences, University of Alberta, Edmonton, AB T6G2E9, Canada; lfbarret@ualberta.ca

**Keywords:** bacterial pathogenesis, Salmonella Typhi, typhoid fever, interbacterial antagonism, type VI secretion systems, T6SS, contact-dependent growth inhibition, immunity genes, comparative genomics

## Abstract

Typhoid fever is a major global health problem and is the result of systemic infections caused by the human-adapted bacterial pathogen *Salmonella enterica* serovar Typhi (*S*. Typhi). The pathology underlying *S*. Typhi infections significantly differ from infections caused by broad host range serovars of the same species, which are a common cause of gastroenteritis. Accordingly, identifying *S*. Typhi genetic factors that impart functionality absent from broad host range serovars offers insights into its unique biology. Here, we used an in-silico approach to explore the function of an uncharacterized 14-gene *S*. Typhi genomic islet. Our results indicated that this islet was specific to the *S. enterica* species, where it was encoded by the Typhi and Paratyphi A serovars, but was generally absent from non-typhoidal serovars. Evidence was gathered using comparative genomics and sequence analysis tools, and indicated that this islet was comprised of Type VI secretion system (T6SS) and contact-dependent growth inhibition (CDI) genes, the majority of which appeared to encode orphan immunity proteins that protected against the activities of effectors and toxins absent from the *S*. Typhi genome. We herein propose that this islet represents an immune system that protects *S*. Typhi against competing bacteria within the human gut.

## 1. Introduction

Typhoid fever is a major health problem in the developing world and is a life-threatening disease that results from systemic infections by the bacterial pathogen *Salmonella enterica* serovar Typhi (*S*. Typhi). *Salmonella* infections are first established in the intestinal tract, typically following consumption of contaminated food or water. Upon initial infection of the gastrointestinal tract, *S*. Typhi infections are generally subclinical, as *S*. Typhi does not stimulate significant levels of intestinal inflammation [1,2]. Maintaining a small immunological footprint at this stage is thought to be important for *S*. Typhi’s subsequent systemic spread via the lymphatic system and bloodstream, triggering the onset of typhoid fever, a disease characterized by a high and prolonged fever and a range of variable symptoms and complications, some of which are life-threatening [1,2]. A small percentage of those infected with *S*. Typhi incur long-term, typically asymptomatic infections that can last decades; these “carriers” can intermittently shed large numbers of *S*. Typhi and are thought to be critical for disease transmission [3,4,5]. *S*. Typhi is a human-restricted pathogen and its virulence and disease properties are similar to other human-restricted *S. enterica* serovars, most notably Paratyphi A [6,7,8]. Paratyphi B and Paratyphi C are also human-adapted serovars reported to cause a typhoid-like disease, but these lineages are scarce, and little is known about their virulence mechanisms [9,10]. These typhoidal serovars are distinct in many important ways compared to the other ~2500 known *S. enterica* serovars, many of which can also infect humans. Broad host range non-typhoidal *S. enterica* serovars such as Typhimurium and Enteritidis are also an important global health issue and are amongst the most common causes of food poisoning worldwide [11]. However, the pathogenesis of these serovars bears little resemblance to that of the typhoidal serovars. Unlike *S*. Typhi, non-typhoidal serovars such as Typhimurium cause short-term infections that generally remain confined to the intestinal tract, where they replicate high numbers and generate significant intestinal inflammation that results in diarrhea, stomach pain, nausea, and other symptomology associated with these infections [12,13]. Deciphering the molecular mechanisms that confer the typhoidal serovars with their unique virulence properties is a fundamental and longstanding goal in the study of *Salmonella* pathogenesis.

Over the past ~20 years there has been an increasing appreciation for the important role that competition with the resident gut microbiota plays in enteric infections. In order to establish a foothold in the intestines, *Salmonella* must effectively compete with the dense population of resident microbes for space and essential nutrients [14,15]. *S*. Typhimurium has been shown to utilize multiple strategies to compete with resident microbiota, including both direct methods and indirect methods that operate by manipulating host immune defenses [16]. A key mechanism by which *S*. Typhimurium overcomes colonization resistance is through eliciting intestinal inflammation, as inflammatory conditions are generally restrictive to commensal bacteria, but provide *S*. Typhimurium with access to key nutrients that support its growth [17,18,19,20,21,22]. It has also been demonstrated that *S*. Typhimurium can directly antagonize the growth of commensal bacteria via its Type VI Secretion System (T6SS), which animal models of infection indicate is important for its ability to colonize the gut [23,24]. T6SS are large, multi-component, proteinaceous molecular machines that inject specific proteins, dubbed effectors, directly into neighbouring cells [25,26,27]. Effectors targeting eukaryotic cells have been identified, but most T6SS function as weapons for interbacterial competition by delivering toxic effectors into adjacent bacteria [28]. In order to avoid self-intoxication, effectors are encoded adjacent to immunity proteins that subvert their toxic effects [25]. *S*. Typhi has also been shown to encode a functional T6SS, yet it does not stimulate significant levels of intestinal inflammation, indicating that the mechanisms by which it competes with the microbial flora differ from those of non-typhoidal serovars [29]. Although there is evidence that microbiome composition influences the outcome of *S*. Typhi infection [30], little is currently known about *S*. Typhi’s interactions with the human microbiota. Improving our understanding of this topic would provide insights into an important but poorly understood aspect of *S*. Typhi pathogenesis and could be used to guide the development of probiotic preventative measures to combat typhoid fever.

The complete genome sequences of *S*. Typhi and *S*. Typhimurium were unveiled nearly 20 years ago, providing the genetic blueprints for *S*. Typhi’s phenotypic differences [31,32]. Analysis of *S*. Typhi genomic loci that are absent from *S*. Typhimurium has been a fruitful avenue for identifying new and important biology. A prime example of this is the typhoid toxin islet, originally identified as an intriguing *S*. Typhi locus comprised of homologs of the pertussis toxin and cytolethal distending toxin subunits [32]. This locus has subsequently been demonstrated to encode a unique A-B type exotoxin dubbed typhoid toxin, an important *S*. Typhi virulence factor that appears to mediate certain disease properties of typhoid fever [33,34,35]. More recently, a comparative genomic approach identified a gene homologous to the typhoid toxin delivery subunit whose phylogenetic distribution tracks with that of the typhoid toxin locus; this finding fueled the discovery that *S*. Typhi produces two different versions of typhoid toxin that have distinct functionality [36]. Here, we exploit expanding genomic databases and bioinformatic toolsets to shed light on the function of an uncharacterized *S*. Typhi genomic islet that is generally absent from non-typhoidal serovars. Our results indicate that this islet is comprised of genes related to T6SS and contact-dependent growth inhibition (CDI) systems, the majority of which appear to encode immunity genes for effectors/toxins that are absent from the *S*. Typhi genome. These results indicate that typhoidal salmonellae have evolved distinct mechanisms to mediate interbacterial competition.

## 2. Results

### 2.1. Identification and Phylogenetic Distribution of an Uncharacterized Genomic Islet Predominantly Encoded by Typhoidal Salmonellae

**Figure 1:**
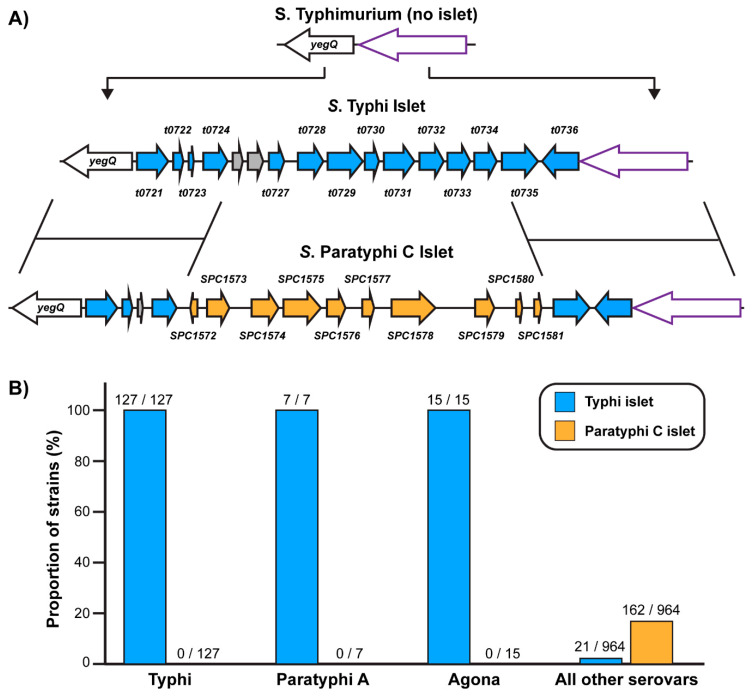
The structure and distribution of genomic islets at the *yegQ* locus of *S. enterica*. In a minority of *S. enterica* strains, a multi-gene islet is inserted upstream of the *yegQ* gene; there are two predominant versions of this islet, which we have dubbed the “Typhi islet” and the “Paratyphi C islet”. (**A**) Gene diagrams showing the architectures of the Typhi and Paratyphi C islets and their insertion sites relative to lineages such as the Typhimurium serovar that lack an islet at this locus. Arrows with purple outlines denote a conserved gene of unknown function that is upstream of the islet insertion site. The Typhi islet is comprised of 14 genes of unknown function (gene identifiers *t0721*-*t0736* in the Ty2 genome) shown using blue arrows. The Paratyphi C islet includes homologs of five of these genes (*t0721, t0722*, *t0724* on one flank, *t0735*-*6* on the other), but does not encode the eight intervening genes and instead encodes ten distinct genes (orange arrows) in their place. *t0723*, which encodes a putative 32 amino acid protein in *S*. Typhi, is a pseudogene in the *S*. Paratyphi C islet; pseudogenes are shown using grey arrows. (**B**) Distributions of the Typhi and Paratyphi C islets amongst sequenced *S. enterica* strains in the NCBI nr database. The percentage of strains of the serovars shown that encode each version of the islet are plotted and the raw numbers (strains with that islet/total strains) are provided above each bar.

Comparison of the first sequenced *S*. Typhi and *S*. Typhimurium genomes revealed that the Typhi serovar encodes ~600 genes that are absent from the Typhimurium genome [32]. However, a significant proportion of these genes are associated with putative prophage and ~150 such genes are confined to *Salmonella* Pathogenicity Island 7 (SPI-7), an established *S*. Typhi virulence locus which includes the genes necessary to produce the Vi capsular polysaccharide [37]. Beyond these elements, there are few large, multi-gene *S*. Typhi loci absent from the *S*. Typhimurium genome. Accordingly, we were intrigued by an ~8.5 Kbp *S*. Typhi genomic islet containing 14 putative genes of unknown function that is absent from the *S*. Typhimurium genome (Figure 1A). This islet is inserted between the *yegQ* gene and a gene of unknown function that encodes a putative ~700 amino acid protein (*t0737* in the *S*. Typhi Ty2 genome, *STM2135* in the *S*. Typhimurium LT2 genome); this is henceforth referred to as the *yegQ* locus. The islet is comprised of genes *t0721*-*t0736* in the *S*. Typhi Ty2 genome (*sty2349*-*sty2364* in the *S*. Typhi CT18 genome) and was previously identified as a genomic element conserved in the Typhi and Paratyphi A serovars but rare in other salmonellae [7], and was noted in a largescale analysis of horizontally-acquired *Salmonella* genes as a locus with an unusually high A+T content that is specific to the Typhi/Paratyphi A lineage [38]. Outside of these observations, to the best of our knowledge, this islet remains completely uncharacterized. We reasoned that the recent expansion of DNA sequence databases and bioinformatic toolsets could provide an avenue to identify a putative function for this islet, which in turn could offer valuable insights into the unique virulence and ecology of typhoidal salmonellae.

We first performed an analysis of the phylogenetic distribution of the *S*. Typhi Ty2 genomic islet by searching the NCBI nonredundant nucleotide (nt) DNA sequence database for organisms that encode similar DNA elements. Thresholding for sequences that align over >50% of the islet, this search unveiled a total of 170 hits, each of which represented a *S. enterica* strain that encodes a similar or identical islet at the *yegQ* locus (Table S1). Importantly, a full-length islet that is at least 99.9% identical to that of the Ty2 strain is encoded by each of the 127 sequenced *S*. Typhi strains represented in the nt database, indicating that this islet is extremely well conserved in the Typhi lineage (Figure 1B, Table S1). Each representative strain of the Paratyphi A and Agona serovars was also found to encode an islet that is >96% identical to the Typhi islet, indicating that it is a core component of the genomes of these serovars as well. Outside of these three serovars, this islet is only found in 21 of 964 sequenced *S. enterica* strains in this database (two of which appear to be Typhi strains that have not been serotyped) and several of these encode truncated versions of the islet (Figure 1B, Table S1). These data indicate that the genomic islet under investigation here is a conserved feature of the Typhi and Paratyphi A serovars but is generally absent from the genomes of nontyphoidal salmonellae.

Our search also identified many other bacterial strains that have DNA sequences with significant similarity to the Typhi islet, but that fell below the 50% query coverage threshold applied above. Outside of the *Salmonella* genus, these hits were limited to strains that encode a homolog of an individual gene encoded by this islet (described further below). However, numerous *S*. *enterica* strains represented in the nr database were found to harbor sequences with clear homology (~95% identity) to >1 kb segment at both ends of the islet. Further investigation revealed that these strains encode a related genomic islet at the *yegQ* locus that overlaps with the Typhi islet at either end (spanning the regions encoding *t0721-t0724* on one end and *t0735-t0736* on the other), but that has a distinct intervening segment encoding 10 putative genes absent from the Typhi islet (Figure 1A). Although this second islet has a broad but sporadic distribution within the enterica subspecies, a surprisingly high proportion of these islets are truncated or contain numerous pseudogenes. To serve as a representative strain, we identified *S*. Paratyphi C (strain RKS4594) as encoding an apparently intact version of this second islet variant [10]. Overall, we found that ~15% of all *S. enterica* strains represented in the nt database encode a version of the Paratyphi C islet (Figure 1B). Taken together, our phylogenetic analysis indicates that at the *yegQ* locus: (i) the majority of salmonellae lack an islet, (ii) the Typhi and Paratyphi A serovars share a conserved islet rarely found in non-typhoidal serovars, and (iii) a related islet that shares several genes is sporadically distributed in an assortment of non-typhoidal strains of the enterica subspecies.

### 2.2. Conserved Domain Analysis Indicates the Uncharacterized Genomic Islet May be Involved in Interbacterial Antagonism

To explore the function of the *S*. Typhi genomic islet, we first took an in-silico approach to seek out potential biological activities of its putative ORFs. Since the Typhi and Paratyphi C islets share several genes, we reasoned that they likely have a related biological function and thus expanded our analysis to include the genes specific to the Paratyphi C islet. We first performed literature searches focused on each gene within these islets but were unable to identify any published data that provided functional insights. We next analyzed each of the 24 putative proteins (5 shared proteins, 9 Typhi islet-specific proteins, 10 Paratyphi C islet-specific proteins) by searching for conserved domains using the NCBI Conserved Domain Database (Table 1) [39]. For the majority of proteins analyzed, including 12 of the 14 putative proteins encoded by the Typhi islet, no conserved domains were identified. Conserved domains were detected in seven of the putative proteins, one of which (Paratyphi C islet protein SPC1581) was found to possess a transposase-associated domain and three others (t0736/SPC1582, SPC1576, SPC1578) provided no functional insights since the domain(s) identified are of unknown function or are found in proteins with diverse, unrelated functions (Table 1). Interestingly, the conserved domains identified for the remaining three proteins all have functions associated with interbacterial antagonism. Specifically, the Paratyphi C islet-specific genes *SPC1575* and *SPC1577* have a domain architecture consistent with a role in T6SS function, including an N-terminal Rhs repeat domain (SPC1575) observed for many T6SS effectors and a PAAR domain (SPC1577) associated with the expelled tip of the T6SS spike that pierces the bacterial envelope to enable effector delivery (Table 1) [25,28]. Additionally, domain analysis indicated that the Typhi islet gene *t0728* encodes a protein related to a CDI system immunity protein produced by *E. coli* O157:H7, strain EC869 (Table 1) [40]. Analogous to T6SS, CDI systems are protein toxin delivery systems used to intoxicate neighboring bacterial cells as a mechanism of interbacterial competition. CDI systems are encoded by three-gene loci comprised of: (i) CdiA, a large protein consisting of a conserved, filamentous N-terminal region that protrudes from the producing bacterium and a variable C-terminal toxin domain that is cleaved and delivered into target cells in a receptor-dependent manner, (ii) CdiB, an outer membrane protein required for CdiA export, and (iii) CdiI, an immunity protein that neutralizes the activity of the CdiA toxin to protect against self-intoxication [41]. Interestingly, other than *t0728*, *S*. Typhi does not appear to encode other CDI genes, indicating that *t0728* likely represents an orphan immunity gene (*cdiI* gene) with the capacity to protect *S*. Typhi against competing bacteria that encode EC869-like CDI toxins. Taken together, these results suggest that the genomic islet under investigation has a function related to interbacterial antagonism.

### 2.3. Homologs of S. Typhi Genomic Islet Genes are Found in T6SS/CDI Gene Clusters in Diverse Bacteria

**Figure 2:**
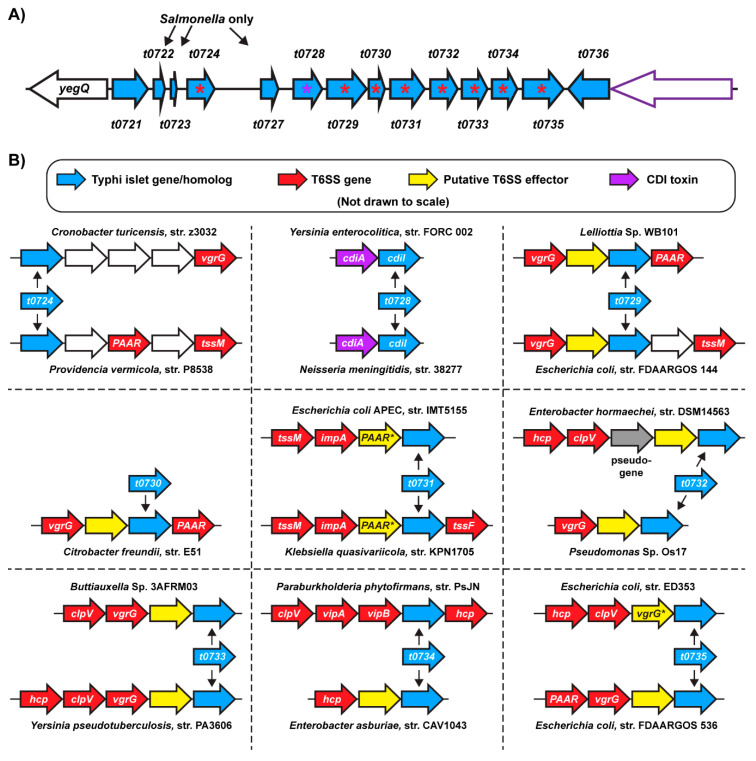
Homologs of most *S*. Typhi genomic islet genes are commonly found within T6SS loci. Homologs of the putative proteins encoded by the *S*. Typhi genomic islet at the *yegQ* locus were identified using tBLASTn. The genomic locus of the identified homologs was then analyzed, revealing that homologs from most *S*. Typhi islet genes are found in T6SS gene clusters. (**A**) Gene diagram depicting the *S*. Typhi genomic islet under investigation, showing the genes that have homologs encoded within T6SS loci (red asterisk) and contact-dependent inhibition (CDI) system loci (purple asterisk). BLAST searches did not identify homologous proteins for t0722, t0723 and t0727 outside of the *Salmonella* genus. (**B**) Gene diagrams showing the local genomic context of homologs of Typhi islet genes that are encoded within T6SS gene clusters. Homologs from a representative strain from two distinct lineages/genome contexts are shown; for *t0730*, homologs were only identified in a single T6SS gene cluster that was limited to the *Citrobacter* genus. Data supporting the identification of homologs can be found in Table S2, data supporting T6SS and CDI gene annotations can be found in Table S3 and data supporting the putative T6SS effector annotations can be found in Table S4. Gene sizes not to scale in (**B**).

Genes involved in T6SS and CDI systems are generally organized into discrete, multi-gene loci, many of which are found on horizontally-acquired genomic islands or associated with mobile genetic elements [26,41]. Accordingly, we hypothesized that if the *S*. Typhi genomic islet is comprised of genes with a function related to these systems, we might be able to identify homologs encoded within T6SS or CDI gene clusters. We therefore searched for homologs of each of the putative *S*. Typhi islet ORFs outside of the *Salmonella* genus, mapped the genomic context of the identified homologs and then used conserved domain analysis to identify the function of neighboring genes (Figure 2, Tables S2 and S3). Searches for t0722, t0723, and t0727 did not identify any homologs outside of the *Salmonella* genus (Figure 2A) and searches for the putative ORFs on either end of the islet, t0721 and t0736, revealed that homologs can be found at the *yegQ* locus (in the absence of a genomic islet) in other γ-proteobacteria (Figure S1). Notably, the Typhi islet has a very low A+T content of 34% suggesting a foreign evolutionary source, but the A+T content of *t0721* and *t0736* is ~50% and does not differ substantially from the *Salmonella* genomic average. Together, these data suggest that while *t0721* and *t0736* were likely co-acquired by *S*. Typhi with the remaining islet genes, they likely have a distinct evolutionary source and function. Consistent with the domain analysis above, *t0728* homologs could be found downstream of putative *cdiA* genes, further supporting the hypothesis that *t0728* represents an orphan CDI immunity gene (Figure 2B, Table S3). Importantly, homologs of each of the remaining eight *S*. Typhi islet ORFs were found in T6SS gene clusters encoded by assorted proteobacterial strains (Figure 2B). For the majority of *S*. Typhi islet genes, we were able to identify homologs in T6SS loci that had different synteny, indicating that homologs are found in multiple distinct T6SS gene clusters. Surprisingly, we did not identify any instances where homologs of two different Typhi islet genes were found within the same locus or even within the same genome, suggesting that the Typhi islet is comprised of T6SS-related genes that are functionally-independent. Collectively, these data imply that the *S*. Typhi genomic islet under investigation here represents a mosaic of ancillary T6SS and CDI genes.

### 2.4. Comparative Genomics and Hidden Markov Model (HMM) Homology Searches Indicate that the S. Typhi Genomic Islet is Primarily Comprised of Orphan Immunity Proteins

Analysis of many diverse T6SS loci has unveiled conserved features of their genomic organization. For example, T6SS effector proteins are often encoded immediately downstream of *hcp* or *vgrG* genes, presumably because their association with these proteins can be essential for their delivery [25,42,43]. Similarly, an extraordinarily well-conserved feature of T6SS effector proteins is that they are paired in a bicistronic fashion with a cognate immunity protein that resides immediately downstream [28,44]; this conserved synteny is also observed with the CDI system [41]. In the above analysis, we noted many instances where homologs of Typhi islet genes were found two genes downstream of a *vgrG* or *hcp* gene, with an intervening gene that is not a conserved structural component of the T6SS. Given the conserved synteny described above, this indicated to us that many Typhi islet homologs reside downstream of T6SS effectors, suggesting they are immunity proteins. To investigate this hypothesis, we used conserved domain analysis and HMM homology searches to explore the function of the gene residing immediately upstream of the Typhi islet homologs identified in Figure 2 (Table S4, Table 2). For genes residing immediately upstream of t0729, t0730, t0732, and t0733 homologs, our analysis identified conserved enzymatic domains associated with established T6SS effector proteins [44,45,46,47,48,49,50,51,52]. Additionally, genes residing immediately upstream of t0731 and t0735 homologs contain conserved PAAR and VgrG domains respectively with a C-terminal extension of unknown function. This arrangement is characteristic of ‘evolved PAAR’ and ‘evolved VgrG’, well-known classes of T6SS effector proteins in which a C-terminal effector domain is fused to an expelled component of the T6SS spike as an effector delivery mechanism [53,54]. In a separate instance, we identified a gene residing upstream of a *t0735* homolog identified by HMM searches as related to *E. coli* MepA, which exhibits a peptidoglycan endopeptidase activity shared by established T6SS effectors [55]. Finally, a *t0734* homolog was found to reside downstream of a protein with a conserved L,D-transpeptidase domain, suggesting it is a peptidoglycan-active protein. Although this activity in itself is not generally bacteriolytic, a recent report identifies a novel class of peptidoglycan amidase T6SS effectors with L,D-carboxypeptidase activity that exhibits significant similarity to L,D-transpeptidases [56]. Coupled with its location immediately downstream of *hcp*, this suggests that this putative L,D-transpeptidase-domain protein is also likely to encode an effector protein. In all, our analyses identified a putative antibacterial T6SS effector gene or CDI toxin gene immediately upstream of a homolog of eight of the ORFs in the Typhi islet (Figure 2, Table 2).

To further explore the possibility that many Typhi islet genes encode T6SS immunity proteins, we performed HMM homology searches for these proteins against the Protein Data Bank (PDB) database using the HHPred HMM-HMM comparison tool (Table S5). Consistent with our domain and genome localization analyses, this search identified significant homology between t0728 and the EC869 CdiI protein [40], further supporting our proposition that this represents an orphan CDI immunity protein. HHPred analysis also identified significant homology between t0730 and *S*. Typhimurium Tai4, a T6SS immunity protein that protects against the activity of the Tae4 peptidoglycan amidase effector protein (Table 2 and Table S5) [47,48,49]. This is consistent with our finding that a *t0730* homolog is encoded downstream of a putative Tae4 in certain *Citrobacter* strains (Figure 2, Table S4). Finally, our search identified significant homology between t0732 and XAC2610, a characterized *Xanthomonas* immunity protein that protects against a peptidoglycan-degrading effector protein delivered by a Type IV Secretion System (Table 2 and Table S5) [57]. This is congruent with our identification of *t0732* homologs that reside downstream of putative peptidoglycan-degrading T6SS effectors. Collectively, these analyses provide evidence that the eight gene array spanning *t0728*-*t0735* is comprised of orphan immunity proteins to protect against the activities of T6SS effectors or CDI toxins absent from the *S*. Typhi genome. On the basis of the results present here, we propose that the *S*. Typhi genomic islet at the *yegQ* locus be renamed SAIDI-1 (*Salmonella* acquired interbacterial defense islet 1).

## 3. Discussion

Since the first *Salmonella* genomes were published, genetic factors that differentiate typhoidal and non-typhoidal salmonellae have been the subject of intense scrutiny. Despite being quickly recognized as a locus associated specifically with typhoidal salmonellae [7,38], SAIDI-1 long evaded functional prediction or characterization. In the present study, we provide evidence that this islet is primarily comprised of an array of immunity proteins that confer protection against the activities of foreign CDI or T6SS effector proteins. Given this function, it is not surprising that SAIDI-1 evaded characterization, as immunity proteins generally lack readily identifiable sequence elements and domain architectures [26,28]. Our ability to identify a putative function for this islet relied extensively on recent experimental findings expanded genomic databases and bioinformatic resources not available when SAIDI-1 was first identified. This study therefore highlights the merit of revisiting longstanding genomic questions armed with a contemporary bioinformatic toolset.

Our analysis found evidence that 8 of the 14 annotated ORFs in the *S*. Typhi SAIDI-1 are immunity proteins. It is noteworthy that many of the effectors against which these orphan immunity proteins offer protection are active against the bacterial cell wall, with only t0728 protecting against an effector/toxin with a different target (genomic DNA). Consistent with this, only t0728 was predicted to have a cytoplasmic localization using the LocTree3 subcellular localization prediction tool [58], with an extra-cytoplasmic localization predicted for the remaining putative immunity proteins. Of the six annotated ORFs in SAIDI-1 for which a putative function was not identified, A+T content analysis and comparative genomics suggest that the genes on either end of this islet, *t0721* and *t0736*, may have been inherited by *S*. Typhi (perhaps via homologous recombination) as a consequence of their localization at the *yegQ* locus in other lineages, and that their function and evolutionary source is likely distinct from the other SAIDI-1 genes. Three other putative ORFs (*t0722*, *t0723*, and *t0727*) appear to be confined to the *S. enterica* species. We speculate they may not represent bona fide and intact ORFs. Homology searches using the t0727 protein sequence identified putative *Salmonella* proteins with a C-terminal region that is >90% identical to the N-terminal ~50 amino acids of t0727 (e.g., NCBI accession EBE1550624) that contain putative Rhs repeat domains (TIGR03696, E value 6.8 × 10^−33^). Similarly, the DNA sequence of *t0726*, a pseudogene that resides upstream of *t0727*, is homologous to a putative protein encoded by a number of sequenced isolates of the salamae subspecies of *S. enterica* (e.g., AXC83657 from strain SA20011914) that also contain Rhs repeat conserved domains (TIGR03696, E value 1.3 × 10^−31^). In light of their genomic context, this suggests that *t0726* and *t0727* likely represent degraded remnants of T6SS effector proteins. *t0722* and *t0723* both encode very small putative proteins (65 and 32 amino acids respectively) and given our inability to identify homologs outside this *Salmonella* islet, it is possible that these too represent remnants of degraded proteins. Finally, although *t0724* homologs are encoded within T6SS loci in other bacterial species, our analysis did not shed any light on its specific function. We therefore propose that the 8-gene putative immunity protein array spanning *t0728* through *t0735* is likely the evolutionary driving force underlying the maintenance and conservation of SAIDI-1 in typhoidal salmonellae. Interestingly, this segment of the islet is also the segment that is absent from the Paratyphi C islet, with the exception of *t0735*, which is present in both islets. We therefore propose that the function (or at least the specific activities) of SAIDI-1 is distinct from the related Paratyphi C islet described here.

Orphan T6SS and CDI immunity proteins have been identified in diverse bacterial lineages and have been shown to play important roles in mediating competition with closely-related bacteria that inhabit the same ecosystem [59,60,61,62,63]. Importantly, this includes residents of the mammalian gut, where “acquired interbacterial defence” (AID) clusters, comprised of arrays of orphan T6SS immunity genes, are widespread amongst diverse *Bacteroides* species and play a key role in shaping interactions between related species or strains [60,63]. Humans are thought to be the principal environmental reservoir for *S*. Typhi and its evolution is thought to have been largely directed by its association with this host. We therefore propose that, analogous to the characterized *Bacteriodes* AIDs, the function of SAIDI-1 is to provide defense against antagonistic mechanisms of closely-related bacteria in the human gut. This hypothesis is supported by the nature of the strains we identified that encode homologs of SAIDI-1 genes, the majority of which are enteric proteobacteria. The present study therefore suggests that the AID immune systems recently identified in commensal bacteria are also employed by a human pathogen to defend against interbacterial antagonism in the human gut.

Presently, it is not clear why SAIDI-1 is predominantly found in human-adapted, typhoidal lineages of *Salmonella*. One possible explanation is that the specific effectors/toxins against which SAIDI-1 offers protection are commonly encountered within the human gut but are encountered less frequently in other *Salmonella* host species. In this scenario, SAIDI-1 would provide a selective advantage for typhoidal serovars, but the evolutionary costs of maintaining SAIDI-1 would outweigh the benefits for other serovars, the majority of which are generalists that infect a wide range of hosts. Another intriguing possibility is that SAIDI-1 is important for the unique transmission of typhoidal serovars. Humans are the only known environmental reservoir for these pathogens, and persistently-infected, asymptomatic carriers are thought to be crucial for their transmission [3,4,5]. Carriers intermittently shed high numbers of *S*. Typhi in “showers” that are thought to be initiated when *S*. Typhi re-enters the intestinal tract from the gallbladder (its presumed site of chronic carriage in most cases) via bile ducts and is subsequently excreted in feces [5]. Although the large numbers of *S*. Typhi excreted during transmission indicate that *S*. Typhi undergoes extensive replication, it is unknown when and where this growth occurs. The circumstances and location(s) of this replication therefore might offer unique competitive challenges from commensal bacteria for which SAIDI-1 might confer a selective advantage. In any scenario, identifying the ecologically-relevant organisms that produce the effectors against which SAIDI-1 confers protection would provide a potential mechanism to identify species/strains within human microbiota that are important competitors for *S*. Typhi.

In summary, we have identified a putative interbacterial defense system encoded on a genomic islet found predominantly in the genomes of typhoidal salmonellae, which we have named SAIDI-1. We provide evidence that numerous genes encoded within this islet are orphan immunity proteins that counteract the activities of T6SS effectors and CDI system toxins encoded by other enteric proteobacteria. We propose that SAIDI-1 represents an immune system that protects *S*. Typhi against the attacks of commensal bacteria that compete with *S*. Typhi within the human gut.

## 4. Materials and Methods

### 4.1. Phylogenetic Distribution Analyses

To analyze the distribution of the *S*. Typhi genomic islet at the *yegQ* locus, the complete *S*. Typhi strain Ty2 islet sequence, defined as the inserted sequence in this genome relative to the *S*. Typhimurium strain LT2 genome, was used as a query for BLASTn searches against the NCBI nonredundant nucleotide (nt) database. This database includes complete genomic sequences for thousands of bacterial strains including more than a thousand *S. enterica* strains from dozens of different serovars. Strains containing a Typhi islet were defined as sharing >70% sequence identity over >50% of the query sequence. All islets that met these criteria had >93% sequence identity, >68% query coverage, and an E value of 0. To determine whether these islets were encoded at the *yegQ* locus, a second analogous BLASTn search was performed that included ~1 kbp flanks of the core genome on either end of the islet, seeking sequences that did not continuously align over the islet boundaries.

A second class of islet was identified as a series of hits from our original searches that represented *Salmonella* genomic sequences with high (>90%) sequence identity to the Typhi islet, but that only aligned over ~40% of the sequence. Representative sequences from these genomes were analyzed and compared and their genomic locus was determined, revealing that they represented a second islet variant at the *yegQ* locus. BLASTn searches analogous to those described above for the Typhi islet revealed that each of these sequences represented a related islet (“Paratyphi C” islet) encoded at the *yegQ* locus. These analyses revealed that there is considerably more variation in the Paratyphi C-type islets, generally in the form of truncation and degradation (pseudogenes) within the segment of the islet that is distinct from the Typhi islet. When analyzing the distribution of the Paratyphi C islets, degraded islets were categorized as Paratyphi C islets if they significantly aligned over >40% of the Paratyphi C RKS4594 islet, including at least 500 bp of the region absent from the Typhi version of the islet. The putative ORFs encoded by the Paratyphi C islet were identified by a combination of prior annotation and ORF analysis [64].

### 4.2. HMM Homology Searches and Conserved Domain Analyses

Conserved domain searches were done using the NCBI conserved domain database (CDD), which analyzes input protein sequences seeking statistically significant hits to NCBI curated protein domains as well as those from the SMART, Pfam, COGs, TIGRFAMs, and PRK databases [39]. Hits with an E value <0.01 were considered to be significant. In instances where multiple related domains were identified (typically similar entries from different source databases), domains with the most specific connection to T6SS biology are presented. HMM homology searches were conducted using the HHpred HMM-HMM comparison tool, part of the MPI Bioinformatics Toolkit [65,66]. Searches were done using local alignment mode against the Protein Data Bank (PDB) database (filtered for sequences with <70% identity) seeking homology to structurally-characterized proteins [67]. Identified hits with an E value <0.01 were considered to be significant.

### 4.3. Genomic Context Analysis for S. Typhi Genomic Islet Homologs

To identify homologs of the putative proteins encoded by the Typhi genomic islet, tBLASTn searches of the nt database were performed (BLOSUM 62, word size 3) filtering for sequences from bacteria (taxid:2) and excluding sequences from the *Salmonella* genus (taxid:690). Only genomes encoding protein sequences with a query coverage >70% and E value <0.0001 were considered. The genome contexts of homologs that met these criteria were then analyzed individually by identifying putative ORFs encoded within an apparent operon (continuous series of genes encoded on the same strand as the identified homolog with <1 kbp spaces between each gene). ORFs were then analyzed using conserved domain analysis (described above) to infer a putative function. For the nine genes shown in Figure 2, this analysis revealed that the majority of homologs were encoded within operons in which multiple ORFs have putative functions associated with T6SS (or CDI systems, for *t0728*). Two representative examples were selected for each of these nine genes for Figure 2 by prioritizing: (i) clear T6SS-related functionality of neighboring genes, (ii) distinct synteny in the two examples, indicating that they represent different T6SS gene clusters, and (iii) maximal evolutionary distance between the two selected examples. Putative T6SS effectors were identified in these gene clusters using HMM homology searches and conserved domain analyses, coupled with literature searches seeking experimental evidence connecting the identified enzymatic domains/homologs to characterized T6SS effector proteins.

## Figures and Tables

**Table 1:** Conserved domains identified in putative ORFs within the Typhi and Paratyphi C genomic islets.

Gene	Domains	E Value ^$^	Comment
*t0736/SPC1582* (Both islets)	Tetratricopeptide repeat (TPR) domain (pfam13424)	7.65 × 10^−4^	TPR domains are protein interaction modules found in a broad array of functionally-diverse proteins
*t0728* (Typhi islet only)	CDI inhibitor, EC869-like domain (cd13445)	6.1 × 10^−69^	A conserved domain of immunity proteins that protect against the activity of contact-dependent growth inhibition (CDI) system toxins related to *E. coli* EC869
*SPC1575* (Paratyphi C islet only)	Rhs-repeat core domain (TIGR03696) RhsA domain (COG3209) OCRE domain (cd16074)	5.1 × 10^−31^ 2.7 × 10^−22^ 9.8 × 10^−3^	Rhs repeats are found in toxic bacterial proteins, including certain insecticidal toxins and T6SS-delivered effector proteins OCRE domains have no defined function
*SPC1576* (Paratyphi C islet only)	Tetratricopeptide repeat (TPR) domain (cl34042)	1.6 × 10^−3^	TPR domains are protein interaction modules found in a broad array of functionally-diverse proteins
*SPC1577* (Paratyphi C islet only)	PAAR-like domain (cd14738)	6.7 × 10^−4^	A conserved domain found in the proline-alanine-alanine-arginine (PAAR) repeat superfamily. The domain forms the sharp conical extension of the T6SS VgrG spike
*SPC1578* (Paratyphi C islet only)	PRK09687 domain HEAT repeat domain (COG1413) HEAT repeat domain (pfam13646)	7.2 × 10^−145^ 3.8 × 10^−15^ 3.0 × 10^−8^	PRK09687 is a conserved domain of unknown function HEAT repeats are found in a broad array of functionally-diverse proteins
*SPC1581* (Paratyphi C islet only)	InsA C-terminal domain (cl37596) InsA domain (COG3677)	8.4 × 10^−17^ 4.3 × 10^−6^	InsA domains are associated with IS1-family transposases

^$^E value: The number of hits to the NCBI conserved domain database with scores equal to or better than the given hit that would be expected to occur by random chance. Values < 0.01 were considered to be significant.

**Table 2:** Putative orphan immunity proteins identified in the *S*. Typhi genomic islet.

Gene	Homolog DS of CDI/T6SS Toxin ^$^	Homology to Immunity Protein ^#^	Putative Function *
*t0728*	Yes	Yes	Immunity protein for an EC869-like CDI toxin, which is a Zn^2+^-dependent DNase that degrades genomic DNA.
*t0729*	Yes	Not detected	Immunity protein for a peptidoglycan-degrading T6SS effector protein
*t0730*	Yes	Yes	Immunity protein for a Tae4-like peptidoglycan-degrading T6SS effector
*t0731*	Yes	Not detected	Immunity protein for a T6SS effector of unknown function
*t0732*	Yes	Yes	Immunity protein for a peptidoglycan-degrading T6SS effector protein
*t0733*	Yes	Not detected	Immunity protein for a peptidoglycan-degrading T6SS effector protein
*t0734*	Yes	Not detected	Immunity protein for a peptidoglycan-degrading T6SS effector protein
*t0735*	Yes	Not detected	Immunity protein for a peptidoglycan-degrading T6SS effector protein

^$^: Homolog identified for this gene that is encoded immediately downstream of a putative T6SS antibacterial effector protein or a CDI toxin (Figure 2, Tables S2–S4); ^#^: Gene encodes a putative protein with significant homology to an experimentally-validated immunity protein (Table S5); *: Functional predictions based on described functions of identified immunity protein homologs (Table S5) and/or the predicted activities of putative upstream effector proteins (Table S4).

## Supplementary Materials

The following are available online at https://www.mdpi.com/2076-0817/9/7/559/s1. Supplementary information includes five Tables and one Figure: Table S1: Genomes in the NCBI nt database that encode a genomic islet similar to the *S*. Typhi islet at the *yegQ* locus. Table S2: Homologs of genes encoded within the *S*. Typhi genomic islet identified in Figure 2. Table S3: Evidence supporting the “T6SS gene” and “CDI toxin” annotations from Figure 2. Table S4: Evidence supporting the “putative T6SS effector” annotations from Figure 2. Table S5: Typhi islet genes with homology to established immunity proteins as detected by HMM homology searches. Figure S1: *t0721* and *t0736* homologs are commonly found at the *yeqQ* locus.

## References

[1] Dougan G., Baker S. (2014). Salmonella enterica serovar Typhi and the pathogenesis of typhoid fever. Annu Rev. Microbiol..

[2] Parry C.M., Hien T.T., Dougan G., White N.J., Farrar J.J. (2002). Typhoid Fever. N. Engl. J. Med..

[3] Gunn J.S., Marshall J.M., Baker S., Dongol S., Charles R.C., Ryan E.T. (2014). Salmonella chronic carriage: Epidemiology, diagnosis, and gallbladder persistence. Trends Microbiol..

[4] Gonzalez-Escobedo G., Marshall J.M., Gunn J.S. (2011). Chronic and acute infection of the gall bladder by salmonella Typhi: Understanding the carrier state. Nat. Rev. Microbiol..

[5] Gopinath S., Carden S., Monack D. (2012). Shedding light on salmonella carriers. Trends Microbiol..

[6] The C.S., Chua K.H., Thong K.L. (2014). Paratyphoid fever: Splicing the global analyses. Int. J. Med. Sci..

[7] McClelland M., Sanderson K.E., Clifton S.W., Latreille P., Porwollik S., Sabo A., Meyer R., Bieri T., Ozersky P., McLellan M. (2004). Comparison of genome degradation in Paratyphi A and Typhi, human-restricted Serovars of salmonella Enterica that cause typhoid. Nat. Genet..

[8] Didelot X., Achtman M., Parkhill J., Thomson N.R., Falush D. (2007). A bimodal pattern of relatedness between the salmonella Paratyphi A and Typhi genomes: Convergence or divergence by homologous recombination. Genome Res..

[9] Selander R.K., Beltran P., Smith N.H., Barker R.M., Crichton P.B., Old D.C., Musser J.M., Whittam T.S. (1990). Genetic Population Structure, Clonal Phylogeny, and Pathogenicity of Salmonella Paratyphi B. Infect. Immun..

[10] Liu W.Q., Feng Y., Wang Y., Zou Q.H., Chen F., Guo J.T., Peng Y.H., Jin Y., Li Y.G., Hu S.N. (2009). Salmonella Paratyphi C: Genetic Divergence From Salmonella Choleraesuis and Pathogenic Convergence With Salmonella Typhi. PLoS ONE.

[11] Majowicz S.E., Musto J., Scallan E., Angulo F.J., Kirk M., O’Brien S.J., Jones T.F., Fazil A., Hoekstra R.M. (2010). The global burden of Nontyphoidal salmonella gastroenteritis. Clin. Infect. Dis..

[12] Grassl G.A., Finlay B.B. (2008). Pathogenesis of enteric salmonella infections. Curr. Opin. Gastroenterol..

[13] Haraga A., Ohlson M.B., Miller S.I. (2008). Salmonellae interplay with host cells. Nat. Rev. Microbiol..

[14] Yurist-Doutsch S., Arrieta M.C., Vogt S.L., Finlay B.B. (2014). Gastrointestinal microbiota-mediated control of enteric pathogens. Annu. Rev. Genet..

[15] Stecher B., Hardt W.D. (2011). Mechanisms controlling pathogen colonization of the gut. Curr. Opin. Microbiol..

[16] Anderson C.J., Kendall M.M. (2017). Salmonella Enterica Serovar Typhimurium strategies for host adaptation. Front. Microbiol..

[17] Bäumler A.J., Sperandio V. (2016). Interactions between the microbiota and pathogenic bacteria in the gut. Nature.

[18] Stecher B., Robbiani R., Walker A.W., Westendorf A.M., Barthel M., Kremer M., Chaffron S., Macpherson A.J., Buer J., Parkhill J. (2007). Salmonella Enterica Serovar Typhimurium exploits inflammation to compete with the intestinal Microbiota. PLoS Biol..

[19] Behnsen J., Jellbauer S., Wong C.P., Edwards R.A., George M.D., Ouyang W., Raffatellu M. (2014). The cytokine Il-22 promotes pathogen colonization by suppressing related commensal bacteria. Immunity.

[20] Winter S.E., Thiennimitr P., Winter M.G., Butler B.P., Huseby D.L., Crawford R.W., Russell J.M., Bevins C.L., Adams L.G., Tsolis R.M. (2010). Gut inflammation provides a respiratory electron acceptor for salmonella. Nature.

[21] Thiennimitr P., Winter S.E., Winter M.G., Xavier M.N., Tolstikov V., Huseby D.L., Sterzenbach T., Tsolis R.M., Roth J.R., Bäumler A.J. (2011). Intestinal Inflammation Allows Salmonella to Use Ethanolamine to Compete With the Microbiota. Proc. Natl. Acad. Sci. USA.

[22] Rivera-Chávez F., Winter S.E., Lopez C.A., Xavier M.N., Winter M.G., Nuccio S.P., Russell J.M., Laughlin R.C., Lawhon S.D., Sterzenbach T. (2013). Salmonella uses energy taxis to benefit from intestinal inflammation. PLoS Pathog..

[23] Brunet Y.R., Khodr A., Logger L., Aussel L., Mignot T., Rimsky S., Cascales E. (2015). H-NS silencing of the salmonella pathogenicity island 6-Encoded Type VI secretion system limits salmonella Enterica Serovar Typhimurium Interbacterial killing. Infect. Immun..

[24] Sana T.G., Flaugnatti N., Lugo K.A., Lam L.H., Jacobson A., Baylot V., Durand E., Journet L., Cascales E., Monack D.M. (2016). Salmonella Typhimurium Utilizes a T6SS-mediated antibacterial weapon to establish in the host gut. Proc. Natl. Acad. Sci. USA.

[25] Ho B.T., Dong T.G., Mekalanos J.J. (2014). A view to a kill: The bacterial type VI secretion system. Cell Host Microbe.

[26] Silverman J.M., Brunet Y.R., Cascales E., Mougous J.D. (2012). Structure and regulation of the Type VI Secretion system. Annu. Rev. Microbiol..

[27] Cianfanelli F.R., Monlezun L., Coulthurst S.J. (2016). Aim, load, fire: The type VI secretion system, a bacterial Nanoweapon. Trends Microbiol..

[28] Russell A.B., Peterson S.B., Mougous J.D. (2014). Type VI secretion system effectors: Poisons with a purpose. Nat. Rev. Microbiol..

[29] Wang M., Luo Z., Du H., Xu S., Ni B., Zhang H., Sheng X., Xu H., Huang X. (2011). Molecular characterization of a functional type VI secretion system in salmonella Enterica Serovar Typhi. Curr. Microbiol..

[30] Zhang Y., Brady A., Jones C., Song Y., Darton T.C., Jones C., Blohmke C.J., Pollard A.J., Magder L.S., Fasano A. (2018). Compositional and functional differences in the human gut microbiome correlate with clinical outcome following infection with wild-type salmonella Enterica Serovar Typhi. MBio.

[31] McClelland M., Sanderson K.E., Spieth J., Clifton S.W., Latreille P., Courtney L., Porwollik S., Ali J., Dante M., Du F. (2001). Complete genome sequence of salmonella Enterica Serovar Typhimurium LT2. Nature.

[32] Parkhill J., Dougan G., James K.D., Thomson N.R., Pickard D., Wain J., Churcher C., Mungall K.L., Bentley S.D., Holden M.T. (2001). Complete genome sequence of a multiple drug resistant Salmonella Enterica Serovar Typhi CT18. Nature.

[33] Fowler C.C., Chang S.J., Gao X., Geiger T., Stack G., Galán J.E. (2017). Emerging insights into the biology of typhoid toxin. Curr. Opin. Microbiol..

[34] Spanò S., Ugalde J.E., Galán J.E. (2008). Delivery of a salmonella Typhi exotoxin from a host intracellular compartment. Cell Host Microbe.

[35] Song J., Gao X., Galán J.E. (2013). Structure and function of the salmonella Typhi Chimaeric A(2)B(5) Typhoid Toxin. Nature.

[36] Fowler C.C., Stack G., Jiao X., Lara-Tejero M., Galán J.E. (2019). Alternate subunit assembly diversifies the function of a bacterial toxin. Nat. Commun..

[37] Pickard D., Wain J., Baker S., Line A., Chohan S., Fookes M., Barron A., Gaora P.O., Chabalgoity J.A., Thanky N. (2003). Composition, acquisition, and distribution of the vi exopolysaccharide-encoding salmonella Enterica pathogenicity island SPI-7. J. Bacteriol..

[38] Vernikos G.S., Thomson N.R., Parkhill J. (2007). Genetic flux over time in the salmonella lineage. Genome Biol..

[39] Marchler-Bauer A., Derbyshire M.K., Gonzales N.R., Lu S., Chitsaz F., Geer L.Y., Geer R.C., He J., Gwadz M., Hurwitz D.I. (2015). CDD: NCBI’s conserved domain database. Nucleic Acids Res..

[40] Morse R.P., Nikolakakis K.C., Willett J.L., Gerrick E., Low D.A., Hayes C.S., Goulding C.W. (2012). Structural basis of toxicity and immunity in contact-dependent growth inhibition (CDI) systems. Proc. Natl. Acad. Sci. USA.

[41] Ruhe Z.C., Low D.A., Hayes C.S. (2013). Bacterial contact-dependent growth inhibition. Trends Microbiol..

[42] Fitzsimons T.C., Lewis J.M., Wright A., Kleifeld O., Schittenhelm R.B., Powell D., Harper M., Boyce J.D. (2018). Identification of novel Acinetobacter Baumannii Type VI secretion system antibacterial effector and immunity pairs. Infect. Immun..

[43] Cianfanelli F.R., Alcoforado Diniz J., Guo M., De Cesare V., Trost M., Coulthurst S.J. (2016). VgrG and PAAR proteins define distinct versions of a functional Type VI secretion system. PLoS Pathog..

[44] Russell A.B., Hood R.D., Bui N.K., LeRoux M., Vollmer W., Mougous J.D. (2011). Type VI secretion delivers Bacteriolytic effectors to target cells. Nature.

[45] Li L., Zhang W., Liu Q., Gao Y., Gao Y., Wang Y., Wang D.Z., Li Z., Wang T. (2013). Structural Insights on the bacteriolytic and self-protection mechanism of muramidase effector Tse3 in Pseudomonas aeruginosa. J. Biol. Chem..

[46] Wang T., Ding J., Zhang Y., Wang D.C., Liu W. (2013). Complex structure of type VI peptidoglycan muramidase effector and a cognate immunity protein. Acta Crystallogr. D Biol. Crystallogr..

[47] Russell A.B., Singh P., Brittnacher M., Bui N.K., Hood R.D., Carl M.A., Agnello D.M., Schwarz S., Goodlett D.R., Vollmer W. (2012). A widespread bacterial type VI secretion effector superfamily identified using a heuristic approach. Cell Host Microbe.

[48] Zhang H., Zhang H., Gao Z.Q., Wang W.J., Liu G.F., Xu J.H., Su X.D., Dong Y.H. (2013). Structure of the Type VI effector-immunity complex (Tae4-Tai4) provides novel insights into the inhibition mechanism of the effector by its immunity protein. J. Biol. Chem..

[49] Benz J., Reinstein J., Meinhart A. (2013). Structural insights into the effector—Immunity system Tae4/Tai4 from salmonella Typhimurium. PLoS ONE.

[50] Tang B.L., Yang J., Chen X.L., Wang P., Zhao H.L., Su H.N., Li C.Y., Yu Y., Zhong S., Wang L. (2020). A predator-prey interaction between a marine Pseudoalteromonas sp. and Gram-positive bacteria. Nat. Commun..

[51] Weber B.S., Hennon S.W., Wright M.S., Scott N.E., de Berardinis V., Foster L.J., Ayala J.A., Adams M.D., Feldman M.F. (2016). Genetic dissection of the type VI secretion system in acinetobacter and identification of a novel peptidoglycan hydrolase, Tagx, required for its biogenesis. MBio.

[52] de Campos S.B., Lardi M., Gandolfi A., Eberl L., Pessi G. (2017). Mutations in Two Paraburkholderia phymatum Type VI secretion systems cause reduced fitness in Interbacterial competition. Front. Microbiol..

[53] Jana B., Salomon D. (2019). Type VI secretion system: A modular toolkit for bacterial dominance. Future Microbiol..

[54] Wood T.E., Howard S.A., Wettstadt S., Filloux A. (2019). PAAR proteins act as the ’sorting hat’ of the type VI secretion system. Microbiology.

[55] Marcyjaniak M., Odintsov S.G., Sabala I., Bochtler M. (2004). Peptidoglycan amidase MepA is a LAS metallopeptidase. J. Biol. Chem..

[56] de Sousa S.S., Hespanhol J.T., Nicastro G.G., Matsuyama B.Y., Mesnage S., Patel A., de Souza R.F., Guzzo C.R., Bayer-Santos E. (2020). A Superfamily of T6SS Antibacterial Effectors Displaying L,D-carboxypeptidase activity towards peptidoglycan. bioRxiv.

[57] Souza D.P., Oka G.U., Alvarez-Martinez C.E., Bisson-Filho A.W., Dunger G., Hobeika L., Cavalcante N.S., Alegria M.C., Barbosa L.R., Salinas R.K. (2015). Bacterial killing via a Type IV secretion system. Nat. Commun..

[58] Goldberg T., Hecht M., Hamp T., Karl T., Yachdav G., Ahmed N., Altermann U., Angerer P., Ansorge S., Balasz K. (2014). LocTree3 prediction of localization. Nucleic Acids Res..

[59] Zhang D., de Souza R.F., Anantharaman V., Iyer L.M., Aravind L. (2012). Polymorphic toxin systems: Comprehensive characterization of trafficking modes, processing, mechanisms of action, immunity and ecology using comparative genomics. Biol. Direct..

[60] Wexler A.G., Bao Y., Whitney J.C., Bobay L.M., Xavier J.B., Schofield W.B., Barry N.A., Russell A.B., Tran B.Q., Goo Y.A. (2016). Human symbionts inject and neutralize antibacterial toxins to persist in the gut. Proc. Natl. Acad. Sci. USA.

[61] Kirchberger P.C., Unterweger D., Provenzano D., Pukatzki S., Boucher Y. (2017). Sequential displacement of type vi secretion system effector genes leads to evolution of diverse immunity gene arrays in vibrio Cholerae. Sci. Rep..

[62] Ting S.Y., Bosch D.E., Mangiameli S.M., Radey M.C., Huang S., Park Y.J., Kelly K.A., Filip S.K., Goo Y.A., Eng J.K. (2018). Bifunctional immunity proteins protect bacteria against FtsZ-Targeting ADP-Ribosylating toxins. Cell.

[63] Ross B.D., Verster A.J., Radey M.C., Schmidtke D.T., Pope C.E., Hoffman L.R., Hajjar A.M., Peterson S.B., Borenstein E., Mougous J.D. (2019). Human gut bacteria contain acquired Interbacterial defence systems. Nature.

[64] Sequence Manipulation Suite. https://www.bioinformatics.org/sms2/orf_find.html.

[65] Söding J. (2005). Protein homology detection by HMM-HMM comparison. Bioinformatics.

[66] Zimmermann L., Stephens A., Nam S.Z., Rau D., Kübler J., Lozajic M., Gabler F., Söding J., Lupas A.N., Alva V. (2018). A Completely reimplemented MPI bioinformatics Toolkit with a new HHpred server at its core. J. Mol. Biol..

[67] Burley S.K., Berman H.M., Kleywegt G.J., Markley J.L., Nakamura H., Velankar S. (2017). Protein data bank (PDB): The single global macromolecular structure archive. Methods Mol. Biol..

