# Supplementary material for: Identification of A Putative T6SS Immunity Islet in Salmonella Typhi
Source: Pathogens. 2020 Jul 11;9(7):559. doi: 10.3390/pathogens9070559 (PMC7400221; doi:10.3390/pathogens9070559)
Supplement: Supplementary file 1 [file pathogens-09-00559-s001.zip › Supplemental Material.docx]

**Table S2.** Homologs of genes encoded within the *S*. Typhi genomic islet identified in Figure 2

| **Strain** | **Accession Number** | **Query Coverage^#^** | **Identity** | **E-value^$^** |
| --- | --- | --- | --- | --- |
| **t0724** | | | | |
| *C. turicensis,* str. *z3032* | CBA30498.1 | 94% | 34% | 1x10^-17^ |
| *P. vermicola* str. P8538 | QIC16694.1 | 77% | 34% | 3x10^-09^ |
| **t0728** | | | | |
| *Y. enterocolitica* str. FORC 002 | AKF39539.1 | 96% | 48% | 7x10^-38^ |
| *N. meningitidis* str. 38277 | APY28862.1 | 98% | 40% | 1x10^-31^ |
| **t0729** | | | | |
| *Lelliottia* Sp. WB101 | AVY98127.1 | 74% | 53% | 5x10^-53^ |
| *E. coli,* str. FDAARGOS 144 | AVG00440.1 | 75% | 48% | 7x10^-45^ |
| **t0730** | | | | |
| *C. freundii*, str. E51 | QFI26661.1 | 92% | 61% | 1x10^-26^ |
| **t0731** | | | | |
| *E. coli*, str. IMT5155 | AJB36004.1 | 86% | 41% | 6x10^-36^ |
| *K. quasivariicola*, str. KPN1705 | ASV18728.1 | 86% | 45% | 7x10^-42^ |
| **t0732** | | | | |
| *E. hormaechei*, str. DSM14563 | AOQ02131.1 | 94% | 35% | 4x10^-19^ |
| *Pseudomonas* Sp. Os17 | BAQ76586.1 | 83% | 40% | 1x10^-24^ |
| **t0733** | | | | |
| *Buttiauxella* Sp. 3AFRM03 | AYN30308.1 | 100% | 59% | 5x10^-56^ |
| *Y. pseudotuberculosis*, str. PA3606 | AJK15959.1 | 99% | 55% | 5x10^-52^ |
| **t0734** | | | | |
| *P. phytofirmans,* str. PsJN | ACD15358.1 | 87% | 38% | 6x10^-20^ |
| *E. asburiae*, str. CAV1043 | AZL64551.1 | 95% | 32% | 1x10^-16^ |
| **t0735** | | | | |
| *E. coli*, str. ED353 | AHW84725.1 | 91% | 61% | 2x10^-72^ |
| *E. coli*, str. FDAARGOS 536 | AYY95976.1 | 91% | 61% | 3x10^-72^ |

^$^E value: The number of alignments with scores equal to or better than this “hit” expected to occur by random chance. Values < 0.0001 were considered to be significant.

^#^Query coverage: Only homologs that significantly aligned over >70% of the Typhi islet query gene were considered for this analysis

**Table S3.** Evidence supporting the "T6SS gene" and “CDI toxin” annotations from Figure 2.

| **Strain** | **T6SS/CDI**  **protein** | **Accession**  **Number** | **Conserved domains** | **E value^$^** | **Putative function** |
| --- | --- | --- | --- | --- | --- |
| **t0724** | | | | | |
| *C. turicensis*, str. z3032 | VgrG | CBA30506.1 | T6SS VgrG  (pfam13296) | 1.5x10^-14^ | T6SS spike protein |
| *P. vermicola* str. P8538 | PAAR | QIC16692.1 | PAAR Motif  (pfam05488) | 6.7x10^-26^ | T6SS spike tip protein |
|  | TssM | QIC16690.1 | IcmF  (COG3523) | 6.5x10^-124^ | T6SS membrane complex protein |
| **t0728** | | | | | |
| *Y. enterocolitica,* str. FORC 002 | CdiA | AKF39540.1 | Haemagluttinin repeat (pfam13332)  EC869-like Cdi toxin (cd13444) | 3.0x10^-04^  1.1x10^-53^ | CDI system CdiA toxin |
| *N. meningitidis,* str. 38277 | CdiA | APY28863.1 | Haemagluttinin repeat (pfam13332)  EC869-like Cdi toxin (cd13444) | 7.9x10^-38^  2.4x10^-55^ | CDI system CdiA toxin |
| **t0729** | | | | | |
| *Lelliottia* Sp. WB101 | VgrG | AVY98125.1 | T6SS Rhs Vgr (TIGR03361) | 0 | T6SS spike protein |
|  | PAAR | AVY98128.1 | T6SS PAAR (NF033420) | 5.2x10^-47^ | T6SS spike tip protein |
| *E. coli,* str. FDAARGOS 144 | VgrG | AVG00438.1 | Rhs Vgr  (TIGR01646) | 2.3x10^-155^ | T6SS spike protein |
|  | TssM | AVG02835.1 | IcmF (COG3523) | 1.5x10^-114^ | T6SS membrane complex protein |
| **t0730** | | | | | |
| *C. freundii*, str. E51 | VgrG | QFI26663.1 | T6SS Rhs Vgr  (TIGR03361) | 0 | T6SS spike protein |
|  | PAAR | QFI26660.1 | T6SS PAAR (NF033420) | 1.7x10^-52^ | T6SS spike tip protein |
| **t0731** | | | | | |
| *E. coli*, str. IMT5155 | TssM | AJB36001.1 | IcmF (COG3523) | 4.4x10^-114^ | T6SS membrane complex protein |
|  | ImpA | AJB36002.1 | ImpA N-terminal (pfam06812) | 2.0x10^-20^ | T6SS inner membrane protein |
| *K. quasivariicola*, str. KPN1705 | TssM | ASV18731.1 | IcmF (COG3523) | 1.7x10^-113^ | T6SS membrane complex protein |
|  | ImpA | ASV18730.1 | ImpA N-terminal (pfam06812) | 3.7x10^-04^ | T6SS inner membrane protein |
|  | TssF | ASV18727.1 | T6SS TssF (pfam05947) | 0 | T6SS baseplate protein |
| **t0732** | | | | | |
| *E. hormaechei*, str. DSM14563 | Hcp | AOQ00127.1 | T6SS Hcp1 (TIGR03344) | 4.9x10^-64^ | T6SS inner tube protein |
|  | ClpV | AOQ00126.1 | T6SS ClpV1 (TIGR03345) | 0 | T6SS ATPase protein |
| *Pseudomonas* Sp. Os17 | VgrG | BAQ76588.1 | T6SS Rhs Vgr (TIGR03361) | 0 | T6SS spike protein |
| **t0733** | | | | | |
| *Buttiauxella* Sp. 3AFRM03 | ClpV | AYN27052.1 | T6SS ClpV1 (TIGR03345) | 0 | T6SS ATPase protein |
|  | VgrG | AYN27051.1 | Rhs Vgr (TIGR01646) | 1.5x10^-156^ | T6SS spike protein |
| *Y. pseudotuberculosis*, str. PA3606 | Hcp | AJK18260.1 | T6SS Hcp1 (TIGR03344) | 1.4x10^-63^ | T6SS inner tube protein |
|  | ClpV | AJK17138.1 | T6SS ClpV1 (TIGR03345) | 0 | T6SS ATPase protein |
|  | VgrG | AJK16633.1 | Rhs Vgr (TIGR01646) | 0 | T6SS spike protein |
| **t0734** | | | | | |
| *P. phytofirmans,* str. PsJN | ClpV | ACD15361.1 | T6SS ClpV1 (TIGR03345) | 0 | T6SS ATPase protein |
|  | VipA | ACDj15360.1 | T6SS VipA (pfam05591) | 3.9x10^-80^ | T6SS contractile sheath protein |
|  | VipB | ACD15359.1 | T6SS VipB (pfam05943) | 0 | T6SS contractile sheath protein |
|  | Hcp | ACD15357.1 | T6SS Hcp (pfam05638) | 1.1x10^-51^ | T6SS inner tube protein |
| *E. asburiae*, str. CAV1043 | Hcp | AZL64553.1 | T6SS Hcp1 (TIGR03344) | 1.3x10^-55^ | T6SS inner tube protein |
| **t0735** | | | | | |
| *E. coli*, str. ED353 | Hcp | AHW84722.1 | T6SS Hcp1 (TIGR03344) | 9.0x10^-68^ | T6SS inner tube protein |
|  | ClpV | AHW84723.1 | T6SS ClpV1 (TIGR03345) | 0 | T6SS ATPase protein |
| *E. coli*, str. FDAARGOS 536 | PAAR | AYY95565.1 | T6SS PAAR (NF033420) | 2.0x10^-47^ | T6SS spike tip protein |
|  | VgrG | AYY95566.1 | T6SS Rhs Vgr (TIGR03361) | 0 | T6SS spike protein |

^$^E value: The number of hits to the NCBI Conserved Domain Database with scores equal to or better than the given hit that would be expected to occur by random chance. Values < 0.01 were considered to be significant.

**Table S4.** Evidence supporting the "putative T6SS effector" annotations from Figure 2.

| **Strain** | **Accession Number** | **Comment** |
| --- | --- | --- |
| **t0729** | | |
| *Lelliottia* Sp. WB101 | AVY98126.1 | ORF at T6SS locus that encodes a protein with a transglycosylase SLT domain (pfam01464, E value 1.0x10^-03^). There are established antibacterial peptidoglycan hydrolyzing T6SS effector proteins that contain this conserved domain [44-46]. |
| *E. coli,* str. FDAARGOS 144 | AVG00439.1 | ORF at T6SS locus that encodes a protein with a transglycosylase SLT domain (pfam01464, E value 5.4x10^-03^). There are established antibacterial peptidoglycan hydrolyzing T6SS effector proteins that contain this conserved domain [44-46]. |
| **t0730** | | |
| *C. freundii*, str. E51 | QFI26662.1 | ORF at T6SS locus that encodes a protein with a T6SS amidase effector protein 4 (Tae4) domain (pfam14113, E value 1.2x10^-17^). Tae4 is an established antibacterial T6SS effector that cleaves peptidoglycan peptide bonds [47-49]. |
| **t0731** | | |
| *E. coli*, str. IMT5155 | AJB36003.1 | ORF at T6SS locus that encodes a protein with a PAAR domain containing an uncharacterized C-terminal extension (cd14744, E value 5.3x10^-25^). This arrangement is characteristic of ‘evolved PAAR’, a well-known class of T6SS effector proteins [53,54]. |
| *K. quasivariicola*, str. KPN1705 | ASV18729.1 | ORF at T6SS locus that encodes a protein with a PAAR domain containing an uncharacterized C-terminal extension (cd14744, E value 4.4x10^-20^). This arrangement is characteristic of ‘evolved PAAR’, a well-known class of T6SS effector proteins [53,54]. |
| **t0732** | | |
| *E. hormaechei*, str. DSM14563 | AOQ00125.1 | ORF at T6SS locus. HHPred analysis of the protein sequence reveals a significant hit with a secreted Pseudoalterin enzyme from *Pseudoalteromonas* sp. Cf6-2 (E value 4.3x10^-7^). Pseudoalterin is a metalloprotease that degrades the peptide bonds of peptidoglycan, resulting in bacterial cell lysis [50]. |
| *Pseudomonas* Sp. Os17 | BAQ76587.1 | ORF at T6SS locus encodes a protein with a conserved domain from peptidase family M23 (pfam01551, E value 8.7x10^-05^), a domain associated with previously identified peptidoglycan-targeting T6SS effector proteins [51,52]. |
| **t0733** | | |
| *Buttiauxella* Sp. 3AFRM03 | AYN27050.1 | ORF at T6SS locus that encodes a protein with a conserved domain from peptidase family M23 (pfam01551, E value 7.7x10^-04^), a domain associated with previously identified peptidoglycan-targeting T6SS effector proteins [51,52]. |
| *Y. pseudotuberculosis*, str. PA3606 | AJK16821.1 | ORF at T6SS locus that encodes a protein with a conserved domain from peptidase family M23 (pfam01551, E value 6.4x10^-04^), a domain associated with previously identified peptidoglycan-targeting T6SS effector proteins [51,52]. |
| **t0734** | | |
| *E. asburiae*, str. CAV1043 | AZL64552.1 | ORF at T6SS locus that encodes a protein containing an l,d-transpeptidase catalytic domain (pfam03734, E value 1.3x10^-06^), suggesting this is a cell wall-active enzyme [56]. |
| **t0735** | | |
| *E. coli*, str. ED353 | AHW84724.1 | ORF at T6SS locus that encodes a protein containing a VgrG domain (COG3501, E value 1.4x10^-106^) with a C-terminal extension of unknown function. This arrangement is characteristic of ‘evolved VgrG’, a well-known class of T6SS effector proteins [53]. |
| *E. coli*, str. FDAARGOS 536 | AYY95975.1 | ORF at T6SS locus. HHPred analysis of the protein sequence reveals a significant hit with *E. coli* MepA, a peptidoglycan endopeptidase (1TZP, E value 5.8x10^-03^) [55]. |

^$^ E value: The number of hits with scores equal to or better than the given hit that would be expected to occur by random chance. Values < 0.01 were considered to be significant.

**Table S5**: Typhi islet genes with homology to established immunity proteins as detected by HMM homology searches

| **Gene** | **Homolog PDB ID** | **E value^$^** | **Description** |
| --- | --- | --- | --- |
| ***t0728*** | 4ZQW_B | 8.9x10^-36^ | CdiI protein from *E. coli* EC869, a CDI immunity protein that inhibits the function of an associated CdiA protein, a DNase that degrades the genomic DNA of target cells [40]. |
| ***t0730*** | 4J32_B | 1.0x10^-15^ | *S*. Typhimurium protein Tai4, an immunity protein that protects against the T6SS-deleivered Tae4 effector protein, a cell wall hydrolase [47-49]. |
| ***t0732*** | 4QTQ_A | 8.8x10^-17^ | Xanthomonas citri protein XAC2610, a peptidoglycan hydrolase inhibitor protein that provides immunity against the activity of the upstream T4SS-secreted bacteriolytic effector protein [57]. |

^$^ E value: The number of hits with scores equal to or better than the given hit that would be expected to occur by random chance. Values < 0.01 were considered to be significant.


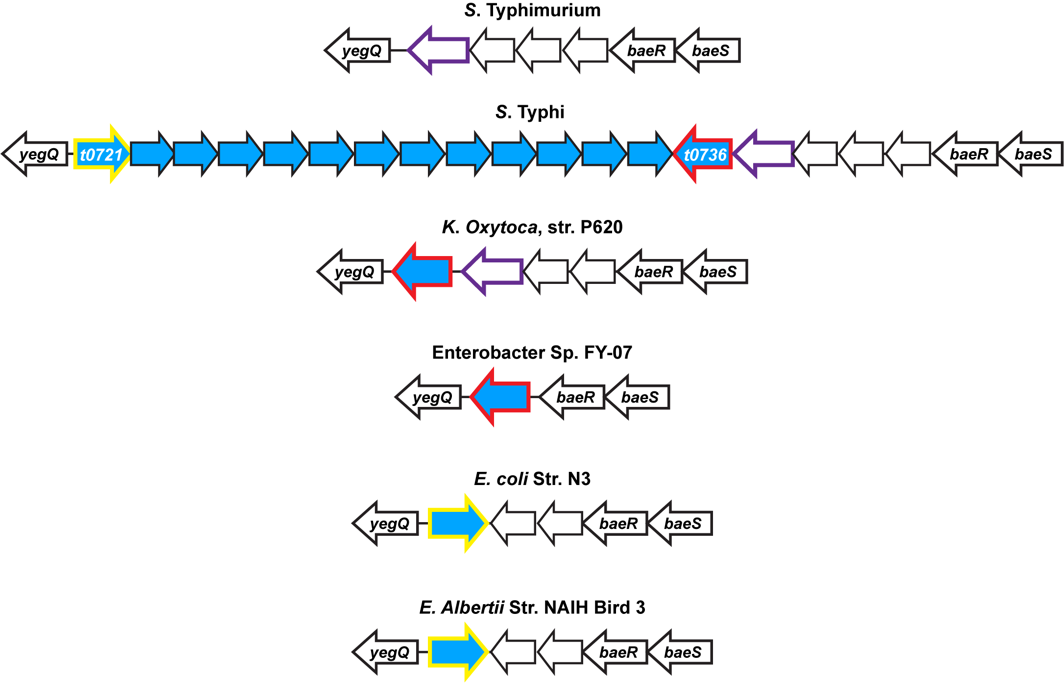


**Figure S1. *t0721* and *t0736* homologs are commonly found at the *yeqQ* locus**. Homologs of the genes at either end of the *S*. Typhi islet (*t0721* and *t0736*), unlike all intervening genes, can be found at the *yegQ* locus in other proteobacterial lineages that lack a genomic islet, suggesting they may be part common components of this genomic locus. Two examples of this phenomenon are depicted using genome diagrams for both *t0721* and *t0736*. For reference, genome diagrams are also shown for the *yegQ* locus of *S*. Typhimurium (*Salmonella* serovar that lacks a *yegQ* islet) and *S*. Typhi. Arrows with a purple outline represent a gene encoded upstream of *yeqQ* in *Salmonella*, arrows with a red outline represent *t0736* homologs, arrows with a yellow outline represent *t0721* homologs. All homologs shown are > 50% identical to the Typhi islet protein over > 70% of the sequence.
